# Risk of breast cancer following exposure to tetrachloroethylene-contaminated drinking water in Cape Cod, Massachusetts: reanalysis of a case-control study using a modified exposure assessment

**DOI:** 10.1186/1476-069X-10-47

**Published:** 2011-05-21

**Authors:** Lisa G Gallagher, Veronica M Vieira, David Ozonoff, Thomas F Webster, Ann Aschengrau

**Affiliations:** 1 Department of Environmental Health Boston University School of Public Health 715 Albany Street, Talbot 4 West, Boston, MA 02118, USA; 2 Department of Epidemiology Boston University School of Public Health 715 Albany Street, Talbot 3 East, Boston, MA 021, USA

## Abstract

**Background:**

Tetrachloroethylene (PCE) is an important occupational chemical used in metal degreasing and drycleaning and a prevalent drinking water contaminant. Exposure often occurs with other chemicals but it occurred alone in a pattern that reduced the likelihood of confounding in a unique scenario on Cape Cod, Massachusetts. We previously found a small to moderate increased risk of breast cancer among women with the highest exposures using a simple exposure model. We have taken advantage of technical improvements in publically available software to incorporate a more sophisticated determination of water flow and direction to see if previous results were robust to more accurate exposure assessment.

**Methods:**

The current analysis used PCE exposure estimates generated with the addition of water distribution modeling software (EPANET 2.0) to test model assumptions, compare exposure distributions to prior methods, and re-examine the risk of breast cancer. In addition, we applied data smoothing to examine nonlinear relationships between breast cancer and exposure. We also compared a set of measured PCE concentrations in water samples collected in 1980 to modeled estimates.

**Results:**

Thirty-nine percent of individuals considered unexposed in prior epidemiological analyses were considered exposed using the current method, but mostly at low exposure levels. As a result, the exposure distribution was shifted downward resulting in a lower value for the 90th percentile, the definition of "high exposure" in prior analyses. The current analyses confirmed a modest increase in the risk of breast cancer for women with high PCE exposure levels defined by either the 90th percentile (adjusted ORs 1.0-1.5 for 0-19 year latency assumptions) or smoothing analysis cut point (adjusted ORs 1.3-2.0 for 0-15 year latency assumptions). Current exposure estimates had a higher correlation with PCE concentrations in water samples (Spearman correlation coefficient = 0.65, p < 0.0001) than estimates generated using the prior method (0.54, p < 0.0001).

**Conclusions:**

The incorporation of sophisticated flow estimates in the exposure assessment method shifted the PCE exposure distribution downward, but did not meaningfully affect the exposure ranking of subjects or the strength of the association with the risk of breast cancer found in earlier analyses. Thus, the current analyses show a slightly elevated breast cancer risk for highly exposed women, with strengthened exposure assessment and minimization of misclassification by using the latest technology.

## Background

Tetrachloroethylene (also called tetrachloroethylene, perchloroethylene, perc or PCE) is a widely used solvent in textile processing, metal degreasing, and dry cleaning. Almost all of the 30,000 dry cleaning establishments in the United States use PCE as a cleaning solvent [1]. PCE can persist in the environment for as little as a few days in surface water to several years in soil and groundwater. It is commonly detected in drinking water supplies and waste sites from improper disposal from industrial operations and small businesses [1,2]. Recent national surveys have found PCE in 11% of tested wells [1] and in 38% of surface water supplies [2]. It is one of the most important chemicals with respect to occupational and environmental exposures.

In a unique exposure scenario, residents in the Cape Cod region of Massachusetts were exposed to PCE in their drinking water from 1968 through the early 1980s. During this period, this solvent was used to apply a vinyl liner to the interior of asbestos-cement drinking water mains. The liner was intended to address unpleasant taste and odor problems related to naturally acidic water corroding the pipes. A slurry of vinyl resin and PCE was hand sprayed onto the inner surface of the pipe. Because PCE is a volatile solvent, it was assumed it would evaporate prior to installation [3]. However in 1980 water samples taken by the Massachusetts Department of Environment Protection (MA DEP) revealed that evaporation had not been complete and PCE in the liner was continuing to leach into the drinking water supply [4]. Approximately 660 miles of asbestos-cement vinyl-lined pipe (ACVL) had been installed throughout Massachusetts by MA DEP estimates, with a large portion on Cape Cod [5]. PCE levels measured in selected water samples in 1980 ranged from 1.5 to 80 μg/l in medium and high-flow pipes and 1,600 to 7,750 μg/l in low-flow pipes [3]. Remediation to the 1980 EPA suggested no adverse response action level ("SNARL") of 40 μg/l was instituted immediately through bleeder valves and regular line flushing [3]. Continued monitoring now ensures levels are below the current maximum contaminant level of 5 μg/l [6].

Exposure to PCE in drinking water occurs by direct ingestion, dermal exposure during bathing, and, because it volatilizes easily, by inhalation during showering, bathing and other household uses. It has been suggested that lipophilic organic solvents like PCE can accumulate in breast tissue and increase the risk of breast cancer [7]. Prior analyses based on data from two existing population-based case-control studies of Cape Cod women indicated an association between high PCE exposure levels and the occurrence of breast cancer [4,8]. The first study, which assessed breast cancer risk among residents of five Cape Cod towns (Barnstable, Bourne, Falmouth, Mashpee, Sandwich) [8,9], suggested small to moderate increases in risk among women in the highest exposure categories but was limited by the small number of cases (n = 258). A second larger study was therefore initiated to include more recently diagnosed cases from women in the original study towns and more exposed women from three additional towns (Brewster, Chatham, and Provincetown) [4]. The second study also found small to moderate increases in risk among women in the highest exposure category when 0 to 15 years of latency were considered [4].

Exposure to PCE in Cape Cod drinking water supplies was unique in that it was not introduced at the water source or during water treatment, but rather at multiple locations throughout the water distribution system. Because ACVL pipes were installed in response to replacement and expansion needs in a town's water system, some entire neighborhoods installed ACVL pipe, while demographically similar neighborhoods installed them only in one street or one section of a street. Thus, neighboring residents could have vastly different exposure levels, presenting a rare opportunity for an epidemiological study with minimal confounding. It also presented several challenges for assessing exposure.

Since PCE levels in water were not systematically measured until 1980, many years after the first ACVL pipes were installed, exposure levels for subjects in our earlier epidemiological studies [4,8] had to be estimated. We used an algorithm developed by Webler and Brown which uses physical properties and experimental data for PCE leaching from the liner and conditions of the water distribution system. The use of the Webler-Brown model required several simplifying and judgment dependent assumptions regarding water flow through the distribution system [10]. Because we were concerned that these simplifying assumptions might entail exposure misclassifications, we modified the original method by using EPANET, a public domain water distribution system model developed by the U.S. Environmental Protection Agency, to model each town's entire water distribution system and more rigorously assess the pattern of PCE contamination. EPANET is entirely automated and does not require human judgment for its estimates. In this paper, we compare exposure estimates from each method and reexamine the risk of breast cancer using EPANET to refine flow estimates. In addition, the current analysis also examines other exposure metrics (e.g., peak, duration) and includes sensitivity analyses on the leaching rate of PCE, another important model parameter. Lastly, we verify our EPANET model by comparing estimates to measured PCE concentration levels in water samples collected when the PCE leaching problem was discovered in 1980.

## Methods

### Study population

This reanalysis uses existing data from two prior case-control studies on PCE-contaminated drinking water and the risk of breast cancer from our group [4,8]. Study participants were permanent residents of eight towns in the Cape Cod region. Incident cases of breast cancer diagnosed from 1983 through 1993 came from the Massachusetts Cancer Registry. Controls were demographically similar women who also lived on Cape Cod from 1983 through 1993. Living controls 64 years old and younger were selected by random digit dialing and controls 65 years old and older were randomly selected from Medicare records. Deceased controls were randomly selected from records of deceased residents of the eight towns provided by the Massachusetts Bureau of Health, Statistics, Research, and Evaluation. The latter two sources ensured efficient identification of elderly and deceased control subjects, so that the control group was comparable in age and vital status to the cases [4,8].

Consent was obtained according to Massachusetts Cancer Registry guidelines and current addresses and telephone numbers were determined using a variety of sources, such as voter registration lists and telephone books. Trained personnel conducted interviews to obtain demographic characteristics, risk factors for breast cancer, occupational exposure to PCE and a 40-year residential history [4,8].

Overall 1,192 cases and 7,869 controls were selected. Subjects were excluded if they could not be located or contacted (87 cases and 1,125 controls), did not meet residential eligibility criteria (31 cases and 4,404 controls), consent could not be obtained from their physician or subject refused to participate (136 cases and 338 controls), or had unknown PCE exposure status (8 cases and 34 controls). The majority of excluded controls were identified using random digit dialing. Additionally, another 666 eligible random digit dial controls were not interviewed after the target number of control interviews was reached. After these exclusions, the prior analyses included 930 cases and 1,302 controls.

The reanalysis with EPANET exposure measures began with this population. Additional subjects were excluded from the reanalysis if residential information necessary for the EPANET exposure calculation was missing (n = 19). Earlier papers have a detailed description of subject selection and enrollment procedures [4,8]. The Institutional Review Boards of Boston University Medical Center and the Massachusetts Department of Public Health approved the current reanalysis.

### Exposure Assessment

The Webler and Brown model, used in our previous studies, estimated PCE exposure by calculating a Relative Delivered Dose (RDD), a proxy measure that is roughly proportional to the mass of PCE delivered through drinking water to the residence [10]. (See Additional File 1 for more details.) The measure is "relative" as the values are not exactly quantified but magnitude related estimates that preserve the exposure ordering of the subjects. Their model has two main components: one component addresses PCE leaching, and a second component addresses water flow. Both require assumptions to simplify the complex configuration of the water distribution systems. The Webler-Brown algorithm uses the configuration, size, age and water flow rate in the ACVL pipe leading to a subject's home during the residency years to calculate the RDD [10].

The water flow component is particularly important because the direction of water flow determines if contaminated water reaches a given residence (i.e., whether or not water reaching the home had at some point flowed through ACVL pipes); and the rate of flow determines the dilution or accumulation of the leaching PCE. During the Webler-Brown model development in the late 1980s, there were no easily applicable methods for computing water flow across an entire water distribution system at arbitrary points, and so Webler and Brown developed a manual technique to model the flow path outward from a residence to the nearest major water pipe. Thus, this technique only took into account a small portion of the water distribution system. It also required human judgment to simplify flow directions in complex geometries. (See Additional File 2 for more details.)

Recently, software programs with published code such as EPANET have become available with the computational ability to model flow behavior in an entire water distribution system [11], enabling us to improve this important aspect of the PCE exposure assessment. Thus, we replaced the Webler-Brown flow model component with that of EPANET for the current reanalysis (see Additional File 3 for details.) The leaching component of the Webler-Brown model was retained by incorporating it directly into the EPANET source code, which is available on the US EPA website. We will refer to the exposure assessment methodologies using the original Webler-Brown model as the manual method. The method using the additional modification of the EPANET code will be referred to as the automated method.

For the automated method, we created computer represented schematics in EPANET of each town's entire water distribution system using Geographic Information System (GIS) maps of land parcels provided by town assessors and digitized paper maps of water distribution systems provided by the town water departments. These schematics included the locations of water sources, pipes (indicating length, diameter and composition), and consumption nodes, points along the pipe where water consumption occurs. The study area is primarily residential so each land parcel was assumed to represent a water user assigned to the closest pipe node, just as in the manual flow model. Historical operating conditions of the water system were assumed, including normal range of system pressure values and sufficient availability of water at well and tank sources to meet water demand. Constant values were used for other system parameters such as elevation that have little variability in these systems. The simulated water flow pattern was a steady-state model of all residents using water at the same time and was assumed to be typical of any given time of day, day of the week, season, and year.

Both the timing and duration of a subject's residence relative to the installation of the ACVL pipe and the initial amount of PCE in the liner determine the RDD estimate. Fickian (first order) diffusion is the basis for the Webler-Brown leaching model, leading to a simple exponential relationship with a rate constant of 2.25 years based on laboratory experiments and theoretical justifications [3]. The model normalized the amount of PCE available to leach during a given year, assuming a constant initial concentration of PCE in the liner and uniform distribution of PCE across the pipe surface [3]. PCE concentrations were assumed never to reach steady-state because water was always flowing. The movement of PCE through the liner was integrated over each pipe's surface area, given its diameter and length in the EPANET schematic. After we modified the open source code, EPANET simulated the instantaneous flow of water through the thousands of pipe segments and estimated the delivered dose of PCE to all system nodes.

To estimate exposures for the breast cancer re-analysis, participants' residential addresses were geocoded to land parcels using Geographic Information System (GIS) software and then matched to a node on the EPANET pipe network. A participant's exposure began when a vinyl-lined pipe was installed adjacent to or upstream from her residence or from an interview determined move-in date to an exposed residence. A participant's exposure ended at the diagnosis or move out year (whichever was earlier) among cases and at a randomly assigned index year or move out year (whichever was earlier) among controls. Index years were assigned to controls based on the distribution of diagnosis years among the cases. We also considered a range of latency periods, times from the causal action of PCE to the diagnosis or index year (0 to 19 years). As in the prior analyses, we calculated cumulative exposure by adding the RDDs at each exposed residence. Additionally, we calculated peak exposure and number of years exposed for each latency period. Prior analyses of personal dose modeling found little variability in bathing and drinking habits in this study population [12], and so we did not incorporate this information into the present exposure assessment.

One advantage of the automated calculation over the manual method is the ability to vary other model parameters. As previously described, Webler-Brown incorporated leaching into their algorithm as an exponential decay relationship (e^-t/R^) where *R *is the estimated leaching rate constant (2.25 yrs) and *t *is time [3,10]. This simplified the complex process of diffusion of PCE through the vinyl liner over time which depends on the water flow rate, temperature, concentration gradient, density and viscosity; characteristics of the liner; and pipe diameter [3]. Because of uncertainties in the leaching rate constant, we also conducted sensitivity analyses of this parameter by varying the leaching rate from very slow to very fast (0.025 to 10 years) and then examined the impact of these changes on exposure values and associations with breast cancer.

### Examination of Exposure Model Characteristics

We obtained another view of the exposure model characteristics by using a small number of available drinking water sample test results in DEP files from 1980. No written protocols for sampling or laboratory analysis were found in DEP files. Some reports and personal communication with research personnel confirmed the likely analytic equipment was a gas chromatograph using heated static head space analysis with a packed column and a Hall electrolytic conductivity detector [13,14].

Unfortunately these historical samples are not completely satisfactory as a "gold standard." They were used only to give a rough indication of where a problem existed and how severe it was. Shortly after these scoping readings were taken, system-wide remediation was begun and subsequent measurements no longer reflected the exposures of the study subjects. While the models (manual and automated) are not actual measurements, they are based on physical principles with parameters estimated from experiments. The scoping measurements, on the other hand, are rough indications of the presence and level of PCE but are subject to measurement error.

We used the results of these historical samples in a recent validation of the manually estimated PCE concentration [13]. We also used them in the current analysis to examine characteristics of the automated method by generating point concentration estimates at the water sampling locations using estimated values for initial concentration of PCE in the pipe and residential water use. The model point concentrations are used to calculate the cumulative or other doses over time (the RDD) in the breast cancer reanalysis but are estimated instantaneous concentrations at the sampling points of the historical water samples. The same measurements were used in both current and prior analyses and were provided by the manufacturer (Johns Manville Corporation) and the Massachusetts Water Resources Authority [15,16]. The earlier validation of the manual method included the results of 88 water samples from nine Massachusetts towns with ACVL water distribution pipe (Barnstable, Bourne, Brewster, Chatham, Falmouth, Provincetown, Sandwich, Plymouth, and Wareham). The two towns of Plymouth and Wareham were included in addition to the seven Cape Cod study towns because they are geographically adjacent to the study area and had a large number of samples available with appropriate documentation. The current analysis includes only 75 (85%) of these samples because sufficient data were not available for EPANET models of Wareham and portions of Plymouth.

In the prior validation of the manual method, we compared measured and modeled PCE concentrations in the water samples according to water distribution, exposure estimation, and sampling characteristics. These characteristics included the magnitude of modeled flow (defined by tertile as low, medium or high), sampling position along pipe (beginning/middle or end), season (spring or autumn), sampling fixture (tap, hydrant or unknown), sampling personnel, town, and pipe installation year (1968-1972, 1973-1976, 1977-1980). Some of these characteristics (town, position, flow, pipe installation year) are explicitly or implicitly included in the model, while others (sampling personnel, season, and geometric complexity) are not. Some may be sources of measurement error in the historical samples.

Despite its drawbacks, obtaining a different view of the exposure models via these measurements produced information that bolstered confidence in the model validity, an important objective given the significance of the exposure. Thus, we have repeated these comparisons for the automated method.

### Data Analysis

Consistent with our previous analyses, the current reanalysis of associations between breast cancer and model-based PCE exposure first compared women who were ever exposed to PCE-contaminated drinking water to women who were never exposed. Women were defined as ever exposed if one or more residences where they lived prior to their diagnosis date (or comparable reference date for controls) were served by an ACVL pipe. We examined four categories of increasing exposure based on the distribution of PCE exposure in control subjects. Low exposure was defined as less than or equal to the median RDD value, while three different versions of high exposure were successively defined as above the median RDD, above the 75^th ^percentile and above the 90^th ^percentile. These exposure levels were determined for each latency period (0, 5, 7, 9, 11, 13, 15, 17 and 19 years) and each leaching rate constant (0.025, 0.75, 2.25, 5 and 10 years). In addition, peak and duration of exposure were analyzed. Peak exposure was also examined in categories defined by the median, 75^th ^percentile, and 90^th ^percentile. Duration of exposure was examined in intervals of 1-5 years, 5-10 years, and greater than 10 years. The referent group for all analyses was always comprised of women who were unexposed during the entire study period.

In addition to examining associations with breast cancer in relation to percentile exposure categories, we conducted smoothing analyses (PROC LOESS in SAS/STAT^® ^software, version 9.1) to evaluate the potential for nonlinear associations between breast cancer and cumulative and peak exposure [17]. We plotted a nonparametric, smoothed function between the logit function and a continuous variable for exposure (RDD). We used a range of smoothing parameters (0.1 to 0.3) to determine if the relationship between the logit function for breast cancer and exposure changed across levels.

The strength of the association between PCE exposure and the occurrence of breast cancer was estimated with exposure odds ratios (OR) and statistical stability was evaluated with 95% confidence intervals. Multiple logistic regression was used to estimate ORs while controlling for covariates by taking the antilog of the beta coefficient for exposure. The current analysis used the same core confounding variables as the prior analyses: age at diagnosis or index year, vital status at interview, family history of breast cancer, personal history of prior breast cancer, age at first live birth or stillbirth, occupational PCE exposure, and study of origin (first study or second expanded study) [4,8]. While subject selection, data collection, demographic characteristics, and risk factors were similar between the two studies [4,8], we controlled for study of origin because of the differing case-control ratios.

We also stratified the results on bottled water use. Several other potential confounders including education, hormone use, and parity were added to the model one at a time but they did not change the core-adjusted estimates by more than 10% and so were not included in the final models. We calculated 95% confidence intervals for the adjusted ORs using the maximum likelihood estimates of the standard errors.

For the exposure model comparisons, Spearman rank correlation coefficients and linear regression analyses were used. When PCE was non-detectable in water samples, half the detection limit of 0.5 μg/L was used in the analysis. As in the prior analyses, the regression models used log_e _transformed PCE concentrations because the data were skewed with a long upper tail. The proportion of the variance (R^2^) explained was obtained from the regression models and p-values were used to describe statistical stability. All analyses were completed using SAS/STAT^® ^software, version 9.1 [17].

## Results

The current analysis is based on 920 cases and 1293 controls. Nineteen subjects from the prior analyses with missing information needed for the EPANET calculation were excluded from the current analyses. The results of the prior analyses with and without these individuals were virtually identical (data not presented).

As previously described [4,8], subjects were predominantly white, over 60 years old, postmenopausal at diagnosis or index year, and had attained an education level of at least 12 years. More cases than controls did not have children, had their first child at a later age, and had a family history of breast cancer. Occupational exposure to PCE (13%), residence near a dry cleaner (<1%), and water consumption and bathing habits were similar among cases and controls. Approximately 22% regularly consumed bottled water and women were equally divided between mostly taking showers (36%), mostly taking baths (27%) or both (36%).

### PCE Exposure and Breast Cancer

#### PCE Exposure Status

In the current exposure assessment, 48.8% of cases (n = 449) and 50.1% of controls (n = 648) were considered ever-exposed (Table 1). Women were never exposed if none of their residences prior to their diagnosis or index year were served by vinyl-lined pipe. This is higher exposure prevalence than the prior manual assessment without EPANET (20.5% of cases and 16.7% of controls). The lower exposure prevalence in the prior analyses was due to the assumption that subject residences not close to a lined pipe had no exposure. As increasing years of latency were considered, the exposure prevalence became lower in the current assessment, since exposures prior to the latency cut-off were not considered exposed. At 19 years latency, only 9.1% of cases and 6.0% of controls were considered exposed in the current analysis.

**Table 1:** PCE exposure history of breast cancer cases and controls

	PCE-exposed cases		PCE-exposed controls	
Latency period (years)	Prior analysis	Current analysis	Prior analysis	Current Analysis
	(n = 930)	(n = 920)	(n = 1302)	(n = 1293)
0	191 (20.5%)	449 (48.8%)	217 (16.7%)	648 (50.1%)
5	154 (16.6%)	399 (43.4%)	163 (12.5%)	552 (42.7%)
7	128 (13.8%)	365 (39.7%)	135 (10.4%)	496 (38.4%)
9	111 (11.9%)	319 (34.7%)	110 (8.4%)	436 (33.7%)
11	86 (9.2%)	281 (30.5%)	83 (6.4%)	376 (29.1%)
13	65 (7.0%)	242 (26.3%)	52 (4.0%)	325 (25.1%)
15	44 (4.7%)	186 (20.2%)	35 (2.7%)	229 (17.7%)
17	21 (2.3%)	130 (14.1%)	21 (1.6%)	141 (10.9%)
19	9 (1.0%)	84 (9.1%)	9 (0.7%)	78 (6.0%)

#### Distribution of PCE Exposure

The RDD distribution in the control subjects was used to determine exposure categories. While control RDD ranges for the manual and automated methods were similar (0.001 to 243.8 for manual and 3.1 × 10^-6 ^to 240.6 for automated), the distributions were considerably different. In particular, the median, 75^th ^and 90^th ^percentile values were 3.6, 15.5, and 41.8 using the manual method and 2.0, 7.1, 19.5 using the automated method (Table 2), reflecting more RDDs with lower values using the automated method. A Wilcoxon signed rank sum test confirmed that the difference was statistically significant (p < 0.0001).

**Table 2:** Distribution of cumulative RDDs among PCE-exposed controls according to latency period

**Prior Analysis**					
				75^th^	**90**^**th**^
Latency	Minimum	Maximum	Median	Percentile	Percentile
0	0.001	243.8	3.6	15.5	41.8
5	0.02	243.2	6.9	17.6	41.7
7	0.05	242.1	6.9	18.2	40.9
9	0.03	239.4	6.4	16.5	38.4
11	0.1	233.0	6.8	18.5	37.3
13	0.1	217.5	10.3	18.9	36.8
15	0.6	200.6	10.3	18.3	49.1
17	1.3	191.6	8.2	21.5	40.6
19	2.6	169.6	13.6	19.8	169.9
**Current analysis**					
				**75^th^**	**90**^**th**^
**Latency**	**Minimum**	**Maximum**	**Median**	**Percentile**	**Percentile**
0	3.1E-06	240.6	2.0	7.1	19.5
5	0.0002	240.2	2.1	8.8	20.6
7	0.0004	239.4	2.4	8.0	19.0
9	0.0005	237.5	2.3	7.5	17.4
11	0.0002	232.9	1.9	6.7	16.1
13	0.0002	222.0	1.4	4.9	12.8
15	0.0002	197.6	1.1	4.6	14.0
17	0.0002	140.0	1.0	3.9	11.4
19	0.0004	73.4	0.6	2.1	9.5

The automated flow assessment method also shifted the distribution of subjects in the exposure categories (Table 3). Only 60.5% (n = 1095) of those considered unexposed according to the manual method in the prior analyses were designated as unexposed using the automated method. Of the remaining 39.5% (n = 715) considered exposed with the automated method, the majority had RDDs that were less than the 75th percentile (n = 595). However 6.6% (n = 120) of those considered unexposed by the manual method were considered highly exposed by the automated method (5.1% were above the 75^th ^percentile and 1.5% were above the 90^th ^percentile). 72% (n = 28) of women considered exposed above the 90^th ^percentile using the manual method remained in that category using the automated method. Overall, subjects tended to shift to higher exposure categories with the automated method.

**Table 3:** Comparison of percentile categories of PCE exposure by assessment method, no latency

		Automated Method, Current Analysis					
	N	Unexposed	**< 50**^**th **^**percentile**	**> 50-75**^**th **^**percentile**	**>75-90**^**th **^**percentile**	**> 90**^**th **^**percentile**	TOTAL
	*Row %*						
	*Col %*						
**Manual Method, Prior Analysis**	**Unexposed**	1095	410	185	92	28	1810
		*60.50%*	*22.70%*	*10.20%*	*5.10%*	*1.50%*	*100%*
		*98.10%*	*74.80%*	*70.10%*	*55.80%*	*23.30%*	-
	**< 50**^**th **^**percentile**	12	123	42	14	6	197
		*6.10%*	*62.40%*	*21.30%*	*7.10%*	*3.00%*	*100%*
		*1.10%*	*22.40%*	*15.90%*	*8.50%*	*5.00%*	-
	**> 50-75**^**th **^**percentile**	3	13	28	31	18	93
		*3.20%*	*14.00%*	*30.10%*	*33.30%*	*19.40%*	*100%*
		*0.30%*	*2.40%*	*10.60%*	*18.80%*	*15.00%*	-
	**>75-90**^**th **^**percentile**	3	0	7	24	40	74
		*4.10%*	*0.00%*	*9.50%*	*32.40%*	*54.10%*	*100%*
		*0.30%*	*0.00%*	*2.70%*	*14.50%*	*33.30%*	-
	**> 90**^**th **^**percentile**	3	2	2	4	28	39
		*7.70%*	*5.10%*	*5.10%*	*10.30%*	*71.80%*	*100%*
		*0.30%*	*0.40%*	*0.80%*	*2.40%*	*23.30%*	-
	**TOTAL**	1116	548	264	165	120	2213
		*100%*	*100%*	*100%*	*100%*	*100%*	

Note: Mutually exclusive categories shown here, but categories are overlapping (nested) in breast cancer risk analysis. Prior analysis had an RDD value of 3.6, 15.5, and 41.8 for the 50^th^, 75^th ^and 90^th ^percentiles. Current analysis had an RDD value of 2.0, 7.1, and 19.5 for the 50^th^, 75^th ^and 90^th ^percentiles. There are 19 subjects that are excluded because current exposure value could not be calculated due to missing data.

Twenty-one subjects considered unexposed by the automated method had been considered exposed using the manual method. This discrepancy was due to particular areas in three water systems (Barnstable, Chatham and Falmouth). In two of these systems, the path of contaminated water using the automated method went in the opposite direction of the path determined by the manual method and so, accordingly, residences were not affected by nearby ACVL pipe. In the third area, two water mains ran parallel on one street and one main was ACVL pipe and the other not. During EPANET modeling by the automated method, we learned from the water department that the ACVL pipe had likely been installed to transport water across the town and there was no direct connection to nearby residences, thereby making them unexposed.

#### Cumulative PCE exposure

Prior epidemiological analyses found that ever-exposed women had a slightly increased risk of breast cancer (ORs, 1.3-1.8 for all latency periods) but these risks were considerably reduced when adjusted for confounders (ORs 1.0-1.3) (Table 4). The current analysis found no increases in the crude odds ratios until 17 and 19 years of latency were taken into account (ORs 1.3-1.4), but the adjusted odds ratios were null for all latent periods (Table 4).

**Table 4:** Association between breast cancer and any exposure to PCE (ever/never)

**Prior Analysis**		
Latency period (years)	Crude OR (95% CI)	Adjusted OR (95% CI)
0	1.3 (1.0-1.6)	1.1 (0.9-1.4)
5	1.4 (1.1-1.8)	1.2 (0.9-1.5)
7	1.4 (1.1-1.8)	1.1 (0.8-1.5)
9	1.5 (1.1-2.0)	1.2 (0.9-1.6)
11	1.5 (1.1-2.1)	1.1 (0.8-1.6)
13	1.8 (1.3-2.7)	1.3 (0.9-2.0)
15	1.8 (1.2-2.9)	1.3 (0.8-2.1)
17	1.5 (0.8-2.7)	1.0 (0.5-1.9)
19	1.5 (0.6-3.7)	1.1 (0.4-2.9)
**Current analysis**		
**Latency period (years)**	**Crude OR (95% CI)**	**Adjusted OR (95% CI)**
0	1.0 (0.8-1.1)	1.0 (0.8-1.2)
5	1.0 (0.8-1.2)	1.0 (0.8-1.2)
7	1.0 (0.9-1.2)	1.0 (0.9-1.3)
9	1.0 (0.8-1.2)	1.0 (0.8-1.2)
11	1.0 (0.9-1.3)	1.0 (0.8-1.2)
13	1.0 (0.8-1.3)	1.0 (0.8-1.2)
15	1.1 (0.9-1.4)	1.0 (0.8-1.2)
17	1.3 (1.0-1.6)	1.0 (0.7-1.3)
19	1.4 (0.9-2.1)	1.0 (0.7-1.4)

Note: Referent group in the prior analysis was comprised of never exposed cases (n = 739) and controls (n = 1,085). The referent group in the current analysis was comprised of never exposed cases (n = 471) and controls (n = 645). Both adjusted analyses controlled for age at diagnosis or index year, vital status at interview, family history of breast cancer, personal history of breast cancer (before current diagnosis or index year), age at first live birth or stillbirth, occupational exposure to PCE, and study of origin.

Prior analyses found small to moderate increases in risk among highly exposed women with exposures above the 75^th ^and 90^th ^percentile when 0-15 years latency was considered (adjusted ORs 1.6-1.9 for >75^th ^and adjusted ORs 1.3-1.9 for >90^th^, Table 5). The current reanalysis found no increased risk for women above the 75^th ^percentile (adjusted ORs 0.9-1.1) and a smaller increases in risk for women above the 90^th ^percentile (adjusted ORs 1.0-1.5). There were more women who were exposed with 17 and 19 years latency in the reanalysis and so there were sufficient numbers of subjects to conduct adjusted analyses for these longer latent periods. The highest adjusted odds ratios for exposures above the 90^th ^percentile were for 5-13 years latency (adjusted OR 1.4-1.5).

**Table 5:** Association between breast cancer and various cumulative PCE exposure

**Prior Analysis**				
	PCE EXPOSURE LEVEL			
Latency period (years)	≤Median	>Median	>75th Percentile	>90th Percentile
0				
Case/control	91/109	100/108	59/54	18/21
COR (95% CI)	1.2 (0.9-1.6)	1.4 (1.0-1.8)	1.6 (1.1-2.3)	1.3 (0.7-2.4)
AOR (95% CI)	1.0 (0.7-1.3)	1.2 (0.9-1.7)	1.6 (1.1-2.4)	1.3 (0.7-2.6)
5				
Case/control	79/82	75/81	50/40	17/16
COR (95% CI)	1.4 (1.0-2.0)	1.4 (1.0-1.9)	1.8 (1.2-2.8)	1.6 (0.8-3.1)
AOR (95% CI)	1.1 (0.8-1.5)	1.3 (0.9-1.8)	1.6 (1.0-2.6)	1.5 (0.7-3.0)
7				
Case/control	59/68	69/67	46/33	17/13
COR (95% CI)	1.3 (0.9-1.8)	1.5 (1.1-2.1)	2.0 (1.3-3.2)	1.9 (0.9-4.0)
AOR (95% CI)	0.9 (0.6-1.3)	1.3 (0.9-1.9)	1.8 (1.1-2.9)	1.7 (0.8-3.6)
9				
Case/control	48/55	63/55	40/27	16/11
COR (95% CI)	1.3 (0.9-1.9)	1.7 (1.2-2.4)	2.2 (1.3-3.6)	2.1 (1.0-4.6)
AOR (95% CI)	0.9 (0.6-1.4)	1.4 (0.9-2.0)	1.9 (1.1-3.2)	1.9 (0.8-4.4)
11				
Case/control	39/42	47/41	29/20	12/8
COR (95% CI)	1.4 (0.9-2.1)	1.7 (1.1-2.6)	2.1 (1.2-3.8)	2.2 (0.9-5.4)
AOR (95% CI)	1.0 (0.6-1.5)	1.4 (0.9-2.1)	1.8 (1.0-3.3)	1.8 (0.7-4.8)
13				
Case/control	35/26	30/26	17/13	8/5
COR (95% CI)	2.0 (1.2-3.3)	1.7 (1.0-2.9)	1.9 (0.9-4.0)	2.3 (0.8-7.2)
AOR (95% CI)	1.4 (0.8-2.3)	1.3 (0.8-2.3)	1.6 (0.7-3.5)	1.7 (0.5-5.2)
15				
Case/control	26/18	18/17	12/8	2/3
COR (95% CI)	2.1 (1.2-3.9)	1.6 (0.8-3.0)	2.2 (0.9-5.4)	1.0 (0.2-5.9)
AOR (95% CI)	1.5 (0.8-2.8)	1.1 (0.6-2.3)	1.7 (0.7-4.3)	-
17				
Case/control	11/11	10/10	4/5	1/2
COR (95% CI)	1.5 (0.6-3.4)	1.5 (0.6-3.5)	1.2 (0.3-4.4)	0.7 (0.1-8.1)
AOR (95% CI)	1.0 (0.4-2.4)	1.0 (0.4-2.6)	0.9 (0.2-3.4)	-
19				
Case/control	6/5	3/4	2/2	0/0
COR (95% CI)	1.8 (0.5-5.8)	1.1 (0.2-4.9)	1.5 (0.2-10.4)	-
AOR (95% CI)	1.3 (0.4-4.2)	0.9 (0.2-4.1)	-	-
**Current analysis**				
	**PCE EXPOSURE LEVEL**			
**Latency period (years)**	<**Median**	**>Median**	**>75th Percentile**	**>90th Percentile**
0				
Case/control	224/324	225/324	123/162	56/64
COR (95% CI)	1.0 (0.8-1.2)	1.0 (0.8-1.2)	1.1 (0.8-1.4)	1.2 (0.8-1.8)
AOR (95% CI)	0.9 (0.8-1.2)	1.0 (0.8-1.3)	1.1 (0.9-1.5)	1.3 (0.9-1.9)
5				
Case/control	193/276	206/276	104/138	53/55
COR (95% CI)	1.0 (0.8-1.2)	1.0 (0.8-1.3)	1.0 (0.8-1.4)	1.3 (0.9-2.0)
AOR (95% CI)	0.9 (0.7-1.2)	1.1 (0.9-1.4)	1.1 (0.8-1.5)	1.4 (0.9-2.1)
7				
Case/control	176/248	189/248	101/124	51/49
COR (95% CI)	1.0 (0.8-1.3)	1.0 (0.8-1.3)	1.1 (0.8-1.5)	1.5 (1.0-2.2)
AOR (95% CI)	1.0 (0.8-1.3)	1.1 (0.9-1.4)	1.1 (0.8-1.5)	1.4 (0.9-2.2)
9				
Case/control	148/218	171/218	89/109	47/43
COR (95% CI)	1.0 (0.7-1.2)	1.1 (0.9-1.4)	1.1 (0.8-1.5)	1.5 (1.0-2.4)
AOR (95% CI)	1.0 (0.7-1.2)	1.1 (0.8-1.3)	1.1 (0.8-1.5)	1.4 (0.9-2.2)
11				
Case/control	122/188	159/188	75/94	40/37
COR (95% CI)	0.9 (0.7-1.2)	1.2 (0.9-1.5)	1.1 (0.8-1.5)	1.5 (1.0-2.5)
AOR (95% CI)	0.9 (0.7-1.2)	1.1 (0.8-1.4)	1.0 (0.7-1.4)	1.4 (0.9-2.4)
13				
Case/control	106/163	136/162	73/81	37/32
COR (95% CI)	0.9 (0.7-1.2)	1.2 (0.9-1.5)	1.2 (0.9-1.7)	1.7 (1.0-2.7)
AOR (95% CI)	0.9 (0.7-1.2)	1.0 (0.8-1.4)	1.1 (0.8-1.6)	1.5 (0.9-2.5)
15				
Case/control	85/115	101/114	50/57	20/22
COR (95% CI)	1.0 (0.8-1.4)	1.2 (0.9-1.6)	1.2 (0.8-1.8)	1.3 (0.7-2.4)
AOR (95% CI)	1.0 (0.7-1.3)	1.0 (0.7-1.3)	1.0 (0.7-1.5)	1.0 (0.5-1.9)
17				
Case/control	61/71	69/70	31/35	18/14
COR (95% CI)	1.2 (0.8-1.7)	1.3 (0.9-1.9)	1.2 (0.7-2.0)	1.7 (0.8-3.5)
AOR (95% CI)	0.9 (0.6-1.3)	1.0 (0.7-1.5)	0.9 (0.5-1.5)	1.3 (0.6-2.7)
19				
Case/control	34/40	50/38	19/19	9/7
COR (95% CI)	1.1 (0.7-1.8)	1.8 (1.1-2.7)	1.3 (0.7-2.6)	1.7 (0.6-4.7)
AOR (95% CI)	0.8 (0.5-1.3)	1.3 (0.8-2.0)	1.0 (0.5-2.0)	1.3 (0.5-3.6)

Note: Referent group in the prior analysis was comprised of never exposed cases (n = 739) and controls (n = 1,085). The referent group in the current analysis was comprised of never exposed cases (n = 471) and controls (n = 645). Both adjusted analyses controlled for age at diagnosis or index year, vital status at interview, family history of breast cancer, personal history of breast cancer (before current diagnosis or index year), age at first live birth or stillbirth, occupational exposure to PCE, and study of origin.

#### Peak PCE exposure

The results for peak exposure were similar when the exposure distribution was divided at the median, 75^th ^and 90^th ^percentile (data not shown). When no latency was taken into account, peak RDD values among the exposed ranged from 1.88E-6 to 85.3; and the median, 75^th ^and 90^th ^percentiles were 0.6, 2.0, 6.0, respectively. The distributions of peak exposure were similar when latency periods were considered. No increases in the risk of breast cancer was seen at exposure levels above the 75^th ^percentile, and small increases in risk was seen at exposure levels above the 90^th ^percentile (adjusted OR 0.9-1.5). The highest adjusted OR was seen when 9 years of latency were considered (adjusted OR = 1.5, 95% CI 0.9-2.3).

#### Duration of PCE Exposure

The analyses of exposure duration found increases in the risk of breast cancer only among women with more than 10 years of exposure when a 13 year latent period was considered (adjusted OR = 1.8, 95% CI, 0.7-4.4) (data not shown). None of the women had more than 10 years exposure duration at longer latent periods. No associations were found between shorter durations of exposure and breast cancer at any latency periods.

#### Sensitivity Analysis for PCE Leaching Rate

Crude and adjusted analyses of the impact of PCE exposure were also conducted for various leaching rates (R) using the automated method. Results for RDDs below the 90^th ^percentile were similar across the different leaching rates (data not shown). A small increased risk of breast cancer was seen among women whose exposure levels were above the 90^th ^percentile across latent periods, including faster leaching rate constants of 0.025 and 0.75 (adjusted ORs 1.0-2.1) as well as slower leaching rate constants of 5.0 and 10.0 (adjusted ORs 1.1-2.4) (see Additional File 4 for more details). The faster leaching rates had a higher RDD cut point for the 90^th ^percentile at longer latencies and fewer subjects exposed above the 90^th ^percentile than the leaching time constant used in the manual method (R = 2.25). The slower leaching rates had a lower RDD cut point for 90^th ^percentile at longer latencies, but similar numbers of exposed subjects as with the leaching constant used in the manual method.

#### Smoothing Analysis to Define PCE Exposure Categories

When we used a range of smoothing parameters to determine if the relationship between the logit function for breast cancer and PCE exposure changed across exposure values, we found similar results across the range of span sizes (data not shown). Using a smoothing parameter of 0.2, the plots for most latency periods showed a slight but steady increase in log odds for RDD values greater than 35. This cut point is most similar to that of the 90^th ^percentile in the prior analyses using the manual method (Tables 2 and 6). When we used an RDD greater than 35 to redefine the highest exposed category, we found 30-40% increases in risk across shorter latent periods (adjusted ORs: 1.3-1.4 for 0-7 years) and 60-100% increases for longer latent periods (adjusted ORs: 1.6-2.0 for 9-15 years, Table 6). The increases for longer latent periods for an RDD greater than 35 were larger than those seen for the 90^th ^percentile in the current analysis, perhaps due to difference in the cut points (Table 6).

**Table 6:** Association between breast cancer and high cumulative PCE exposure: comparison of various cut points

Latency period (years)	Prior Analysis >90th Percentile	Current Analysis >90th Percentile	Current Analysis Smoothing
0			
RDD	41.8	19.5	35
Case/control	18/21	56/64	26/32
COR (95% CI)	1.3 (0.7-2.4)	1.2 (0.8-1.8)	1.1 (0.7-1.9)
AOR (95% CI)	1.3 (0.7-2.6)	1.3 (0.9-1.9)	1.3 (0.7-2.3)
5			
RDD	41.7	20.6	35
Case/control	17/16	53/55	24/30
COR (95% CI)	1.6 (0.8-3.1)	1.3 (0.9-2.0)	1.1 (0.6-1.9)
AOR (95% CI)	1.5 (0.7-3.0)	1.4 (0.9-2.1)	1.3 (0.7-2.3)
7			
RDD	40.9	19.0	35
Case/control	17/13	51/49	23/25
COR (95% CI)	1.9 (0.9-4.0)	1.5 (1.0-2.2)	1.3 (0.7-2.3)
AOR (95% CI)	1.7 (0.8-3.6)	1.4 (0.9-2.2)	1.4 (0.7-2.5)
9			
RDD	38.4	17.4	35
Case/control	16/11	47/43	21/19
COR (95% CI)	2.1 (1.0-4.6)	1.5 (1.0-2.4)	1.6 (0.8-3.0)
AOR (95% CI)	1.9 (0.8-4.4)	1.4 (0.9-2.2)	1.6 (0.8-3.0)
11			
RDD	37.3	16.1	35
Case/control	12/8	40/37	17/14
COR (95% CI)	2.2 (0.9-5.4)	1.5 (1.0-2.5)	1.8 (0.8-3.6)
AOR (95% CI)	1.8 (0.7-4.8)	1.4 (0.9-2.4)	1.8 (0.8-3.9)
13			
RDD	36.8	12.8	35
Case/control	8/5	37/32	13/10
COR (95% CI)	2.3 (0.8-7.2)	1.7 (1.0-2.7)	1.9 (0.8-4.6)
AOR (95% CI)	1.7 (0.5-5.2)	1.5 (0.9-2.5)	2.0 (0.8-4.8)
15			
RDD	49.1	14.0	35
Case/control	2/3	20/22	8/5
COR (95% CI)	1.0 (0.2-5.9)	1.3 (0.7-2.4)	2.1 (0.7-6.6)
AOR (95% CI)	-	1.0 (0.5-1.9)	1.8 (0.6-5.6)
17			
RDD	40.6	11.4	35
Case/control	1/2	18/14	4/5
COR (95% CI)	0.7 (0.1-8.1)	1.7 (0.8-3.5)	1.1 (0.3-4.0)
AOR (95% CI)	-	1.3 (0.6-2.7)	-
19			
RDD	169.9	9.5	35
Case/control	0/0	9/7	0/2
COR (95% CI)	-	1.7 (0.6-4.7)	-
AOR (95% CI)	-	1.3 (0.5-3.6)	-

Note: The referent group was comprised of never exposed cases (n = 471) and controls (n = 645). The adjusted analysis controlled for age at diagnosis or index year, vital status at interview, family history of breast cancer, personal history of breast cancer (before current diagnosis or index year), age at first live birth or stillbirth, occupational exposure to PCE, and study of origin.

The smoothing analyses for peak PCE exposure also showed the same pattern of increasing odds of breast cancer with RDDs greater than 10 over the range of smoothing parameters. Using this cut point to define high peak exposure; the adjusted odds ratios were higher than those using the 90^th ^percentile as the peak exposure cut point (data not shown). Adjusted odds ratios ranged from 1.3 to 2.1 for 0 to 17 years of latency for the new RDD cut point of 10.

#### PCE Exposure and Bottled Water Use

Lastly, we found slight differences in breast cancer risk among ever-exposed women when stratified on regular bottled water use. Ever-exposed women who did not regularly drink bottled water had slightly higher risk (adjusted ORs 1.1- 1.3, 0-19 years' latency) than women who regularly drank bottled water (adjusted ORs 0.6-0.8, 0-19 years' latency). These findings were similar to those of our prior analysis.

### Examination of Exposure Models and Historical Measurements

**Figure 1:**
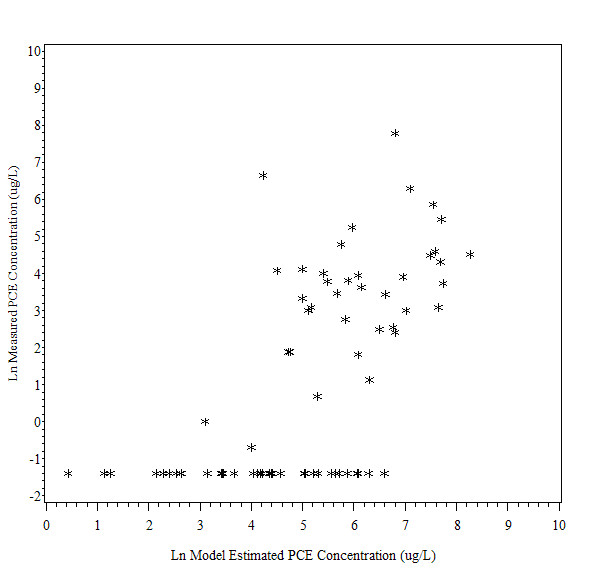
**Comparison of Ln Measured PCE Concentration (ug/L) with Ln Model Estimated PCE Concentration (ug/L): Automated Method**. This figure depicts the relationship between measured PCE concentration and model estimated PCE concentration using the automated method. There was a moderate level of correlation between measured and modeled PCE concentrations using the automated method (Spearman rank correlation coefficient (ρ = 0.65, p < 0.0001).

**Figure 2:**
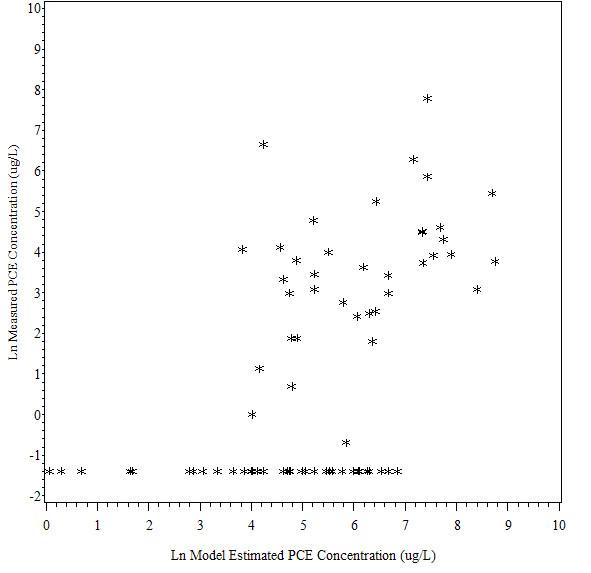
**Comparison of Ln Measured PCE Concentration (ug/L) with Ln Model Estimated PCE Concentration (ug/L): Manual Method**. This figure depicts the relationship between measured PCE concentration and model estimated PCE concentration using the manual method. There was a moderate level of correlation between measured and modeled PCE concentrations using the manual method (ρ = 0.54, p < 0.001) but the magnitude of correlation was lower than that of the automated method.

There was a moderate level of correlation between measured and modeled PCE concentrations using the automated method among the 75 historical water samples (Spearman rank correlation coefficient (ρ = 0.65, p < 0.0001, see Figure 1 and Additional File 5). A lower level of correlation was observed between the measured and PCE concentrations modeled using the manual method (ρ = 0.54, p < 0.001, see Figure 2 and Additional File 5). The latter correlation is slightly higher than that found in the earlier validation of 88 samples (ρ = 0.48, p < 0.0001). When only samples with detectable PCE levels were included, the correlation was weaker using both methods (ρ = 0.38 for automated and 0.39 for manual). Correlations using the automated method were higher in areas that were difficult to model using the manual method's flow assumptions, including locations in complex pipe configurations (ρ = 0.69 vs. 0.49). Results for other characteristics were similar to those from the prior validation (see Additional file 5 for more details).

Slightly higher PCE concentrations were predicted by the manual method, but this difference was small (see Additional File 5 and Figure 2 for details). A simple regression model showed a better fit between the log_e _transformed measured and model-generated PCE concentrations using the automated method (R^2 ^= 0.40, p < 0.0001) compared to manual method (R^2 ^= 0.30, p < 0.0001). This improvement was also observed when only detectable samples were examined (R^2 ^= 0.20, p = 0.005 for automated and R^2 ^= 0.16, p = 0.01 for manual). When stratified regression analyses were conducted according to pipe and sampling characteristics, a better model fit using the automated method was evident for samples taken in complex piping configurations (R^2 ^= 0.47 vs. 0.30), at the middle/beginning of a pipe (R^2 ^= 0.43 vs. 0.27), at hydrants (R^2^= 0.47 vs. 0.21), in areas with higher flow (R^2^= 0.41 vs. 0.22 for medium and 0.26 vs. 0.16 for high), and at the most recently installed pipe (R^2 ^= 0.43 vs. 0.19 for 1977-1980). The automated and manual methods performed similarly in areas with simple configurations, low flow, early installation years (1968-1976), and at pipe ends.

When comparing measured PCE concentrations in all water samples with modeled concentrations using the automated method, the leaching rate constant (2.25 years) used in the manual method resulted in the highest agreement between modeled and measured concentrations. When only samples with detectable levels were included, a faster constant (0.75 years) resulted in modeled values that were slightly better correlated with measured concentrations (ρ = 0.43 vs. 0.38).

## Discussion

Consistent with our earlier findings, the current analyses using an automated method for exposure assessment showed a modest association with breast cancer among highly exposed women. This method identified subjects with low levels of exposure among those previously considered unexposed and identified more highly exposed subjects among those previously considered to have low or moderate exposure levels. Based on comparison with historically measured samples, the automated exposure assessment appears to be more accurate than those derived from the earlier manual method. Thus, exposure misclassification in our prior analyses occurred mainly among subjects with low exposure levels and the current exposure distribution is shifted downward. It was not surprising that comparisons made using the percentile categories of RDD distribution shifted the elevated risks from women whose exposures were above the 75^th ^percentile (in the prior analysis) to women whose exposures were above the 90^th ^percentile (in the current reanalysis). Minimizing exposure misclassification among the unexposed most affected the referent group in the prior analysis, but the increased risk of breast cancer remained among more highly exposed women.

Given the current distribution of exposure, smoothing analyses were an important way to identify a meaningful cut point for "high" exposure. Similar or slightly stronger associations with breast cancer were seen when high exposure was defined at this new cutpoint (RDD > 35). Defining high exposure in the current analysis at an RDD of 35 was most comparable to the 90^th ^percentile RDD in the prior analysis (Table 2). While the majority of subjects classified above the 90^th ^percentile in current analysis also had an RDD above 35 (85-100%, depending on latency), differences in RDD cut points may account for the attenuation of some of the 90^th ^percentile odds ratios.

Because peak and duration of exposure are incorporated in the cumulative exposure measure, all exposure measures were highly correlated. In fact, the Spearman rank correlation coefficient between these measures ranged from 0.93 to 0.99 depending on latency period (p < 0.0001). Thus, while analyses of breast cancer risk using cumulative, peak, and duration of exposure were in good agreement, these analyses could not distinguish effects of intensity or duration of exposure from cumulative exposure.

Depending on the time interval between the installation of an ACVL pipe and the move-in date of a subject, varying the leaching rate should result in different exposure estimates. For example, a subject who moved into a location with ACVL pipe a few years after installation would be unexposed with a fast leaching rate (because the PCE would disappear quickly) or exposed with a slow leaching rate (because the PCE would mostly be remaining). Overall, we found that RDDs were smaller if the leaching rate was faster, and that RDDs were larger if the leaching rate was slower. However, varying the leaching rates did not affect the results of our epidemiological analyses when we used the 90^th ^percentile to define the highest category of exposure because the exposures of the study population still maintained the same rank order.

The manual method's simplified flow estimation was a potential source of exposure misclassification in the prior breast cancer analyses, and we found this to be true for some residences when we compared manual estimates to those with the automated method. The manual method used the tools available at the time and simplified modeling water flow by addressing small sections of the distribution system piping around each residence. Using EPANET to incorporate complex conditions of water flow appears to predict PCE concentrations more accurately compared to measured water samples.

While incorporating EPANET in the exposure model addressed many complexities of the water distribution system that the Webler-Brown flow model could not, some of the same limitations remain. In EPANET, the modeled flow pattern and distribution system conditions were used to represent a wide range of time periods and water usage conditions, but the EPANET assessment still assumed the predicted steady-state flow pattern in the system was typical of any given time of the day, year, or season. Other studies have created more sophisticated models using information on current or historical conditions, such as tank levels, water account data, or pressure data recorded at hydrants, to characterize and validate the EPANET model [18-21]. In the absence of reliable historical data for Cape Cod, we used EPANET to characterize water flow patterns to provide a reasonable method for ranking exposure for subjects in our epidemiological analyses.

In addition, the EPANET assessment did not determine if all land parcels had residences, and therefore water use, during the exposure period. An analysis of one study town (Mashpee) found that limiting water use to parcels occupied in 1980 reduced the number of users by one-half, which changed magnitude of exposure, but did not significantly affect exposure categories because water flow direction remained similar. Nevertheless, other towns with different patterns of development may have different results. Residential build year information could be used to see if improving this aspect of the EPANET model would provide still better exposure information.

Predicting detectable concentrations using measured samples from 1980 was improved with the automated method. Again, this improvement was most evident in areas where applying the manual method was difficult, including complicated configurations, at the middle or initial segment of a pipe, and in higher flow areas. The modeled estimates using the automated method, in addition to incorporating more physical conditions and principles, are better correlated with the water samples from 1980 (p = 0.65 vs. 0.54).

It is important to recognize that there were also likely inaccuracies in the measured concentrations. The water samples were collected in the 1980s to obtain a rough determination of the scope of the PCE leaching problem and begin remediation [5]. Areas with ACVL pipe that were anticipated to have high levels like low flow, dead-end pipes, were preferentially sampled. In the absence of written protocols it is possible there were inconsistencies in water sampling. Aeration from hydrant sampling may have introduced errors depending on sampling procedure and head space in hydrant lines may have caused loss of PCE due to volatilization. Thus, many samples below the detection limit may have been "false negatives," particularly in sample locations in low flow areas near recently installed pipe. Several towns had only one or two samples with detectable levels, suggestive of measurement error, although there are circumstances (e.g. high flow, older pipe) where levels below the detection limit would be expected. Variation in pipe drying times and initial PCE concentrations may have contributed to the low levels.

Testing by the DEP suggested the laboratory's use of head space analysis could also have underestimated PCE levels in the water samples by as much as 80% [13]. A DEP memorandum indicated that this methodology was considered a qualitative, not quantitative, method that required less analysis time than the more accurate purge and trap method and so allowed for a rapid response and remediation [13]. Up to two-fold fluctuations in water concentrations were observed in a sampling study that measured concentrations at the same location and time on consecutive days [14]. Wacholder et al. have used the term "alloyed gold standard" to describe these type of data [22], although the addition of even more sources of measurement error (such as sampling personnel and season) suggest that the historical measurements are not a standard at all but just another view of the data.

As discussed in our prior publication on breast cancer and PCE exposure, this reanalysis is unlikely to be affected by selection bias. The Massachusetts Cancer Registry was the source of all breast cancer cases, and had nearly complete reporting according to a Department of Public Health comparison with other state cancer registries [4]. Demographic characteristics, follow-up and interview rates were similar among cases and controls, and demographic characteristics were similar among participants and non-participants [4,8]. While interviewers were not blinded to a woman's disease status, observation bias was also unlikely to affect the results. The closed-ended questions were carefully written and pre-tested, and interviewers were trained in appropriate interviewing techniques. Also, proxy interviews for deceased cases and controls resulted in comparable information quality [4]. Lastly, the exposure assessments using EPANET were conducted without knowledge of the participant's disease status.

Core confounding variables of age at diagnosis or index year, vital status at interview, family history of breast cancer, personal history of prior breast cancer, age at first live birth or stillbirth, and occupational exposure to PCE were controlled in adjusted analyses. Other potential confounders did not change the core-adjusted ORs by more than 10%, so they were not included in the models. Unmeasured factors, environmental or otherwise, may have resulted in residual confounding, but this is an unlikely explanation for these findings because these factors would need to be strong risk factors for breast cancer and tightly correlated with PCE exposure. The latter is unlikely given the irregular pattern of the ACVL pipe locations.

Animal and epidemiological studies have suggested that PCE exposure is associated with several types of cancer; but in general null effects have been found for breast cancer. Experiments have shown increases in the incidence of liver tumors in mice exposed to PCE orally or by inhalation, and increases in incidence of leukemia and kidney cancer in rats with inhalation exposure [2,23]. There has been no evidence of mammary tumors stemming from PCE exposure in animal assays, although other organic solvents have shown this effect [7,24]. Epidemiological evidence has been provided primarily by occupational studies of dry cleaning workers and people working with solvents in metal industries. Exposure assessments in these studies are difficult, relying on job title or duration of work to define exposure, and so potentially misclassify subjects' exposure. In many studies, there were multiple chemical exposures occurring at the same time, relatively small numbers of women, and missing information on potential confounders. Reviews of the overall weight of evidence, however, prompted the International Agency for Cancer Research (IARC) to classify PCE as a probable human carcinogen and the National Toxicology Program (NTP) to classify PCE as reasonably anticipated to be carcinogenic to humans [23,25].

Studies of the association between PCE and breast cancer incidence have produced inconsistent results. An 11% decreased incidence of breast cancer was seen in a large Scandinavian cohort (n = 23,714), but exposure to PCE was uncertain among this group of laundry and dry cleaning workers [26]. Compared to women in the general population, laundry and dry cleaning workers in a U.S.-Canada population-based study had a lower incidence of breast cancer (mean annual age-standardized rates were, 77.4 vs. 100.3 per 100,000 person-years) [27]. A case-control study in British Columbia examined post-menopausal women whose usual occupation was in dry cleaning industry and found a 4.9-fold increased risk of breast cancer (95%CI 1.3-18.7) after controlling for important risk factors such as family history of breast cancer [28]. Chemically similar to PCE, studies on the solvent trichloroethylene (TCE) and breast cancer show similarly mixed results.

A study of Finnish workers found a decreased risk of breast cancer for women who were biologically monitored for exposure to TCE, PCE and another halogenated hydrocarbon trichloroethane (SIR = 0.84, 95% CI, 0.44-1.48) [29]. A study in Taiwan of electronics factory workers exposed to chlorinated solvents that likely included PCE and TCE found a small but significantly elevated incidence of breast cancer (SIR = 1.19, 95% CI, 1.03-1.36) after adjusting for age and calendar year [30].

Our current analyses made use of technological advances to investigate a limitation of our earlier exposure assessment method and potential source of misclassification. We used GIS software in conjunction with a modification of the open source water distribution modeling software, EPANET, to create a more detailed exposure model that could easily be manipulated for sensitivity analyses and other improvements. EPANET has been used in other epidemiological studies to conduct exposure assessments of distribution system contamination, including simulations to study the extent and severity of source contamination, as well as assess water treatment and system issues such as trihalomethane exposure [18-21,31]. This unique application of EPANET software was made possible by availability of its open source code, which could be adapted for our use, and by GIS software which provided the necessary platform to bring together the different types of data needed to improve the drinking water flow model. In this study, GIS software made it possible to create detailed maps with pipe characteristics, land parcels to represent water users, and participants' residential locations. These maps formed the basis for the EPANET schematics used to simulate water flow and the dispersion of PCE in the exposure model. With this automated method, we were also able to perform sensitivity analyses on the leaching rate.

## Conclusions

Widespread contamination of drinking water by PCE and the limitations of occupational studies made refinement of exposure assessment methods for our Cape Cod studies an important objective. A recent review of published literature confirms that our studies remain the only population-based research on breast cancer in relation to solvent-contaminated drinking water [4,32]. The current analysis shows consistent findings of slightly elevated breast cancer risk for highly exposed women, with strengthened exposure assessment and minimization of misclassification by using the latest technology. Thus, the associations between breast cancer and PCE-contaminated drinking water are relatively robust to refinements in exposure modeling.

## Abbreviations

PCE: Tetrachloroethylene; DEP: Department of Environmental Protection; ACVL: Asbestos-cement vinyl-lined; SNARL: Suggested no adverse response action level; RDD: Relative delivered dose; GIS: Geographic Information Systems; OR: Odds Ratio; AOR: Adjusted odds ratio; COR: Crude odds ratio; CI: Confidence Interval; ND: Not detectable; TCE: Trichloroethylene

## Competing interests

Dr. David Ozonoff is Co-editor-in-Chief of *Environmental Health*. He has recused himself from all decisions involving the acceptance and publication of this manuscript. At the request of the Commonwealth of Massachusetts, in 1980 Dr. Ozonoff was a witness in bankruptcy court in a suit against the Johns-Manville Corporation, manufacturers of the ACVL water mains. He has also, on occasion, testified in personal injury and property damage cases involving exposure to tetrachloroethylene and trichloroethylene. Dr. Aschengrau has served as a consultant in a personal injury case involving chlorinated solvent contamination in the past. None of the parties in any litigation supported, reviewed or had knowledge of this paper. None of the other authors of this study have any competing interests.

## Authors' contributions

LG completed the exposure assessments, conducted statistical analyses, and wrote the initial draft of the manuscript. AA conceived the study, participated in its design and coordination, assisted in the analysis, and finalized the manuscript. DO, TW and VV provided technical input to study design, analysis, modeling, and manuscript preparation. All authors read and approved the final manuscript.

## Supplementary Material

Additional File 1 **Detailed PCE Exposure Calculation**. This file describes the mathematical basis for calculating the PCE exposure measure known as the relative delivered dose (RDD) Click here for file

Additional File 2 **Webler-Brown Flow Model (Manual method)**. This file provides a detailed description of the process for assessing water flow in the Webler and Brown model. Click here for file

Additional File 3 **EPANET Flow Model (Automated Method)**. This file provides a detailed description of the process for assessing water flow using EPANET. Click here for file

Additional File 4 **Results of Sensitivity Analysis for Various PCE Leaching Rates**. This files describes the associations between breast cancer and PCE exposure levels > 90th percentile under various PCE leaching rate assumptions Click here for file

Additional File 5 **Comparison of Exposure Models against Historical Tap Water Measurements**. This file contains detailed results from our validation study which compared the manual (Webler-Brown) and automated (EPANET) exposure models against historical tap water measurements. Click here for file
