# Supplementary material for: Risk of breast cancer following exposure to tetrachloroethylene-contaminated drinking water in Cape Cod, Massachusetts: reanalysis of a case-control study using a modified exposure assessment
Source: Environ Health. 2011 May 21;10:47. doi: 10.1186/1476-069X-10-47 (PMC3125233; doi:10.1186/1476-069X-10-47)
Supplement: Additional File 1 — Detailed PCE Exposure Calculation. This file describes the mathematical basis for calculating the PCE exposure measure known as the relative delivered dose (RDD) [file 1476-069X-10-47-S1.DOCX]

# Additional File 1

**Title: Detailed PCE Exposure Calculation**

The Webler-Brown and EPANET flow models determine the water flow rate which is combined with PCE leaching parameters to calculate the relative delivered dose (RDD) as a measure of PCE exposure. PCE concentrations vary depending on the length of pipe from the source (well or storage tank), the type of pipe it passes through (lined or unlined) and the water flow rate. The RDD is dependent on known pipe characteristics, such as piping configuration, pipe diameter, and age of the ACVL pipe leading to the subject’s home during the residence period. The RDD does not have meaningful units, but rather is a relative measure of the mass of PCE delivered to a home.

To calculate the RDD, pipes are divided into segments (*s*) at nodes (*i*), or points where water use can occur and exposure levels can be calculated over a given time period (*t_1_- t_2_*). As developed by Webler-Brown, a concentration function, *Δ_i_(t)*, for PCE is integrated over the exposure period and multiplied by the household water use (*q*) as shown in the following equation [1, 2]:

The concentration function, *Δ_i_(t)*, is the leaching function of PCE up to node *i* divided by the water flow rate in the pipe leading to it. The leaching function describes the movement of the PCE through the vinyl liner integrated over the pipe surface and normalized to the time of pipe installation. It assumes that there is a finite amount of PCE in the liner, a uniform distribution of the liner along the pipe length, and a uniform starting amount of PCE in the liner. It also assumes that, because water is always flowing, PCE leaches without reaching a steady-state. Based on prior laboratory experiments and theoretical justifications, a model of Fickian diffusion reduces to a simple exponential relationship with a rate constant (*r*) of 2.25 years. The amount of time since the pipe installation date (*t_s_*) determines the amount of PCE available to leach at a given time. The initial concentration (*C_1s_*) and pipe surface area (pipe diameter (*D_s_*) and length (*L_s_*)) also determine the quantity of PCE available to leach [1, 2]. The initial PCE concentration (C_s1_) is assumed to be constant and is dropped during the actual calculation due to its uncertainty.

The second part of the RDD equation addresses the water flow rate leading to a given node (Q_i-1_). It is mathematically shown as the difference between the flow entering the main pipe (Q_o_) and the flow that serves all residences located beyond it. Each residence is represented by the same water flow rate (q). For example, if a pipe has eight residences along it and there are two residences at each node, there are a total of four nodes. The first pipe segment has enough water flow for all eight residences (Q_o_=8q). The flow for next pipe segment is for six residences (6q), the next four residences (4q) and the next two residences (2q). Assumed constants for these parameters were incorporated into the calculation when we converted the RDD to an actual concentration of PCE for our validation study [3]. More details on this calculation are provided in our prior validation study [4]. Prior analyses used the manual Webler-Brown method but improvements in technology enabled us to refine the water flow model using the automated EPANET program.

# References

1. Demond AH: **A Source of Tetrachloroethylene in the Drinking Water of New England: An Evaluation of Toxicity of Tetrachloroethylene and the Prediction of its Leaching Rates from Vinyl-lined Asbestos-cement Pipe.** Massachusetts Institute of Technology, 1982.

2. Webler T, Brown HS: **Exposure to tetrachloroethylene via contaminated drinking water pipes in Massachusetts: a predictive model.** *Arch Environ Health* 1993, **48:**293-297.

3. Massachusetts Water Resources Authority (MWRA): **Drinking Water Test Results. Available: http://www.mwra.state.ma.us/annual/waterreport/metro2003.pdf [accessed 6 June 2005].**

4. Spence L, Aschengrau A, Gallagher L, Webster T, Heeren T, Ozonoff D: **Evaluation of a model for estimating tetrachloroethylene exposure from vinyl-lined asbestos-cement pipes.** *Environmental Health: A Global Access Science Source* 2008, **7**.
