# Supplementary material for: Risk of breast cancer following exposure to tetrachloroethylene-contaminated drinking water in Cape Cod, Massachusetts: reanalysis of a case-control study using a modified exposure assessment
Source: Environ Health. 2011 May 21;10:47. doi: 10.1186/1476-069X-10-47 (PMC3125233; doi:10.1186/1476-069X-10-47)
Supplement: Additional File 2 — Webler-Brown Flow Model (Manual method). This file provides a detailed description of the process for assessing water flow in the Webler and Brown model. [file 1476-069X-10-47-S2.DOCX]

# Additional File 2

# Title: Webler-Brown Flow Model (Manual method)

Webler and Brown developed an approach to determine water flow that we recently improved with EPANET software. The Webler-Brown approach for assessing water flow considered only small, manually manageable sections of a network. A spreadsheet was used to solve simple algebraic equations and the leaching algorithm for the limited set of ACVL pipe segments. Simple geometries of dead-ends and circles were combined to model the direction of water flow. A dead-end was considered a section of pipe with one inlet and no outlet that only served the water users connected. A circle was defined as two dead-ends joined in the middle. This midpoint was considered a point of “no flow” for modeling purposes, although water does actually flow in that segment depending on the number of water users at a given point in time. However, given balanced water use in the area, the water flow at the midpoint, on average, was assumed to be zero [1].

While some areas easily conformed to the compartmental approach of Webler-Brown, it was difficult to apply this approach in settings that did not obviously reduce to simple geometries. To address this limitation, the Webler-Brown model specified several governing assumptions about the flow direction through the distribution system: (1) water flows from larger to smaller diameter pipes; (2) water flows outward from a source; (3) the closest water supply typically dominates and is where flow originates; (4) two or more water mains supplying an area serve equal numbers of homes in that area, as long as distances the water travels are roughly equivalent; and (5) in areas served by two or more water mains, the point where the water meets behaves like a dead end pipe [2].

While these assumptions were appropriate in most situations, their application was limited by the human assessor’s capability to create manageable, accurate segments of the distribution system. Further, the assumptions were difficult to apply in areas with identically sized pipe, particularly when the piping configuration was complex. In addition, while major pipes originating at the source are usually sized larger to carry more water and smaller pipes branch off the larger ones, it is possible for water to flow from smaller to larger pipes, if dictated by water demand and proper pipeline design [3]. The Webler-Brown method also simplified many areas into “circles” and so created presumed “dead-end” flow points at the circle midpoints. However, the true position of these “dead-end” flow points is unknown and is likely to vary considerably when the entire system’s water demand is taken into account. The difficulties in estimating flow patterns and applying these governing assumptions prompted us to incorporate EPANET into our PCE exposure assessments.

1. Webler T, Brown HS: **Exposure to tetrachloroethylene via contaminated drinking water pipes in Massachusetts: a predictive model.** *Arch Environ Health* 1993, **48:**293-297.

2. Webler T, Brown HS: **Appendix A: A User’s Guide to the Delivered Dose Spreadsheet, Cumulative Dose of Tetrachloroethylene received between 1968 and 1985 by Massachusetts Residents from Vinyl Lined Asbestos Cement Water Distribution Pipes: an empirically based model, Final Draft.**; 1989.

3. Mays LW: *Water Distribution Systems Handbook.* McGraw-Hill, New York; 2000.
