# Supplementary material for: Risk of breast cancer following exposure to tetrachloroethylene-contaminated drinking water in Cape Cod, Massachusetts: reanalysis of a case-control study using a modified exposure assessment
Source: Environ Health. 2011 May 21;10:47. doi: 10.1186/1476-069X-10-47 (PMC3125233; doi:10.1186/1476-069X-10-47)
Supplement: Additional File 3 — EPANET Flow Model (Automated Method). This file provides a detailed description of the process for assessing water flow using EPANET. [file 1476-069X-10-47-S3.DOCX]

# Additional File 3

# Title: EPANET Flow Model (Automated Method)

EPANET was originally designed to support water utilities in maintaining and improving their water quality by helping to design sampling and monitoring programs to study disinfection loss and system contamination [1]. For the current study, RDDs and point concentrations were calculated using EPANET. Land parcel and water distribution maps were used to create schematics of water source locations, pipes (indicating length, diameter and composition) and nodes, or points along the pipe where water consumption occurs. Each study residence was assigned to the closest pipe node.

The Webler-Brown method used the water system present in 1988 because assessments were performed manually on available maps from that year. The EPANET method created schematics of the system in 1980, a time closer to the actual exposure period for the subjects in the epidemiological studies. Extensive development of land parcels and addition of large quantities of piping occurred in the 1980s, further supporting the use of the 1980 distribution system with EPANET for our epidemiological studies of historical exposure and cancer risk. Very few assessments were affected by this difference (1988 vs. 1980) because the Webler-Brown method rarely assessed flow in pipes beyond the ACVL pipes which were all installed from 1968 to 1980.

We used typical historical operating conditions and required that modeled pressure at the majority of points fall within a normal operating range (50-70 psi) and not at unrealistically low (<35psi) or high (>100 psi) pressure values. Also, water sources were required to provide sufficient water to all users, either by pumping wells or from gravity-fed storage tanks. Similar to some other studies that have used EPANET, typical and constant values were used for parameters such as pipe resistance coefficients and pipe node elevations [2, 3]. For example, pipe node elevation is dependent on topography which is relatively constant in the Cape Cod area.

Most other studies using EPANET examined contamination from one or a few drinking water sources. The complexity of PCE leaching along multiple pipes throughout a system presented a unique and challenging opportunity to apply this modelling tool. Because EPANET has an open source code, we were able to incorporate the Webler-Brown leaching algorithm directly into EPANET in order to recalculate the RDDs from our prior breast cancer and validation studies. Given an initial concentration of PCE in the liner and the leaching rate, EPANET simulated the instantaneous flow of water through the thousands of pipe segments in each town’s network and estimated PCE leaching at all designated pipes in the system.

1. Rossman LA: **EPANET Users Manual, Version 1.1.**  Risk Reduction Laboratory, Office of Research and Development, USEPA 1994.

2. Aral MM, Maslia ML, Ulirsch GV, Reyes JJ: **Estimating exposure to volatile organic compounds from municipal water-supply systems: use of a better computational model.** *Arch Environ Health* 1996, **51:**300-309.

3. Maslia ML, Sautner JB, Aral MM, Reyes JJ, Abraham JE, Williams RC: **Using water-distribution system modeling to assist epidemiologic investigations.** *Journal of Water Resources Planning and Management-Asce* 2000, **126:**180-198.
