# Supplementary material for: Risk of breast cancer following exposure to tetrachloroethylene-contaminated drinking water in Cape Cod, Massachusetts: reanalysis of a case-control study using a modified exposure assessment
Source: Environ Health. 2011 May 21;10:47. doi: 10.1186/1476-069X-10-47 (PMC3125233; doi:10.1186/1476-069X-10-47)
Supplement: Additional File 4 — Results of Sensitivity Analysis for Various PCE Leaching Rates. This files describes the associations between breast cancer and PCE exposure levels > 90th percentile under various PCE leaching rate assumptions [file 1476-069X-10-47-S4.DOCX]

# Additional File 4

# Title: Results of Sensitivity Analysis for Various PCE Leaching Rates

**Table S1 Association between breast cancer and PCE exposure levels>90th percentile under various PCE leaching rate assumptions**

|  | Leaching Rate | | | | |
| --- | --- | --- | --- | --- | --- |
| Latency period (years) | R=0.025 | R=0.75 | R=2.25 | R=5.0 | R=10.0 |
| 0 |  |  |  |  |  |
| RDD | 25.2 | 18.7 | 19.5 | 22.3 | 20.5 |
| Case/control | 34/37 | 48/59 | 56/64 | 60/61 | 57/62 |
| COR (95% CI) | 1.2 (0.8-2.0) | 1.1 (0.7-1.6) | 1.2 (0.8-1.8) | 1.3 (0.9-1.9) | 1.2 (0.8-1.8) |
| AOR (95% CI) | 1.4 (0.8-2.3) | 1.2 (0.8-1.8) | 1.3 (0.9-1.9) | 1.4 (0.9-2.1) | 1.3 (0.9-1.9) |
| 5 |  |  |  |  |  |
| RDD | 25.2 | 19.2 | 20.6 | 22.3 | 16.0 |
| Case/control | 34/37 | 45/51 | 53/55 | 55/53 | 52/53 |
| COR (95% CI) | 1.2 (0.8-2.0) | 1.2 (0.8-1.8) | 1.3 (0.9-2.0) | 1.4 (0.9-2.1) | 1.3 (0.9-2.0) |
| AOR (95% CI) | 1.4 (0.8-2.3) | 1.2 (0.8-1.9) | 1.4 (0.9-2.1) | 1.4 (0.9-2.1) | 1.3 (0.8-2.0) |
| 7 |  |  |  |  |  |
| RDD | 24.7 | 19.2 | 19.0 | 18.4 | 14.6 |
| Case/control | 35/36 | 44/46 | 51/49 | 51/47 | 49/47 |
| COR (95% CI) | 1.3 (0.8-2.1) | 1.3 (0.8-2.0) | 1.5 (1.0-2.2) | 1.5 (1.0-2.2) | 1.4 (0.9-2.1) |
| AOR (95% CI) | 1.4 (0.9-2.3) | 1.3 (0.8-2.1) | 1.4 (0.9-2.2) | 1.4 (0.9-2.1) | 1.3 (0.8-2.0) |
| 9 |  |  |  |  |  |
| RDD | 24.1 | 19.2 | 17.4 | 15.2 | 10.8 |
| Case/control | 32/34 | 40/40 | 47/43 | 47/41 | 48/41 |
| COR (95% CI) | 1.3 (0.8-2.1) | 1.3 (0.8-2.1) | 1.5 (1.0-2.4) | 1.5 (1.0-2.4) | 1.6 (1.0-2.4) |
| AOR (95% CI) | 1.3 (0.8-2.1) | 1.3 (0.8-2.1) | 1.4 (0.9-2.2) | 1.4 (0.9-2.2) | 1.4 (0.9-2.3) |
| 11 |  |  |  |  |  |
| RDD | 19.2 | 18.1 | 16.1 | 11.4 | 9.2 |
| Case/control | 32/30 | 34/34 | 40/37 | 44/35 | 40/35 |
| COR (95% CI) | 1.4 (0.9-2.4) | 1.3 (0.8-2.2) | 1.5 (1.0-2.5) | 1.7 (1.1-2.7) | 1.5 (1.0-2.5) |
| AOR (95% CI) | 1.4 (0.8-2.3) | 1.3 (0.8-2.1) | 1.4 (0.9-2.4) | 1.5 (0.9-2.5) | 1.4 (0.8-2.2) |
| 13 |  |  |  |  |  |
| RDD | 20.7 | 17.4 | 12.8 | 9.5 | 6.9 |
| Case/control | 26/26 | 29/29 | 37/32 | 37/30 | 33/30 |
| COR (95% CI) | 1.3 (0.8-2.3) | 1.3 (0.8-2.3) | 1.7 (1.0-2.7) | 1.7 (1.0-2.7) | 1.5 (0.9-2.5) |
| AOR (95% CI) | 1.3 (0.7-2.4) | 1.3 (0.7-2.2) | 1.5 (0.9-2.5) | 1.5 (0.9-2.5) | 1.3 (0.7-2.1) |
| 15 |  |  |  |  |  |
| RDD | 20.6 | 17.6 | 14.0 | 9.2 | 5.6 |
| Case/control | 16/19 | 18/21 | 20/22 | 25/21 | 27/21 |
| COR (95% CI) | 1.1 (0.6-2.2) | 1.1 (0.6-2.2) | 1.3 (0.7-2.4) | 1.6 (0.9-2.9) | 1.7 (1.0-3.1) |
| AOR (95% CI) | 1.0 (0.5-2.0) | 1.0 (0.5-1.9) | 1.0 (0.5-1.9) | 1.3 (0.7-2.4) | 1.4 (0.7-2.5) |
| 17 |  |  |  |  |  |
| RDD | 21.1 | 20.3 | 11.4 | 6.5 | 3.8 |
| Case/control | 10/13 | 10/13 | 18/14 | 19/14 | 20/14 |
| COR (95% CI) | 1.0 (0.5-2.4) | 1.0 (0.4-2.4) | 1.7 (0.8-3.5) | 1.8 (0.9-3.7) | 1.9 (1.0-3.8) |
| AOR (95% CI) | 0.8 (0.3-1.8) | 0.7 (0.3-1.8) | 1.3 (0.6-2.7) | 1.4 (0.7-2.9) | 1.5 (0.7-3.1) |
| 19 |  |  |  |  |  |
| RDD | 12.5 | 12.8 | 9.5 | 5.9 | 3.4 |
| Case/control | 10/7 | 10/7 | 9/7 | 9/7 | 10/7 |
| COR (95% CI) | 1.9 (0.7-5.1) | 1.9 (0.7-5.0) | 1.7 (0.6-4.7) | 1.7 (0.6-4.7) | 1.9 (0.7-5.1) |
| AOR (95% CI) | 1.4 (0.5-3.9) | 1.4 (0.5-3.8) | 1.3 (0.5-3.6) | 1.3 (0.5-3.6) | 1.5 (0.5-4.0) |

Note: The referent group was comprised of never exposed cases (n=471) and controls (n=645). The adjusted analyses controlled for age at diagnosis or index year, vital status at interview, family history of breast cancer, personal history of breast cancer (before current diagnosis or index year), age at first live birth or stillbirth, occupational exposure to PCE, and study of origin.
