# Supplementary material for: Risk of breast cancer following exposure to tetrachloroethylene-contaminated drinking water in Cape Cod, Massachusetts: reanalysis of a case-control study using a modified exposure assessment
Source: Environ Health. 2011 May 21;10:47. doi: 10.1186/1476-069X-10-47 (PMC3125233; doi:10.1186/1476-069X-10-47)
Supplement: Additional File 5 — Comparison of Exposure Models against Historical Tap Water Measurements. This file contains detailed results from our validation study which compared the manual (Webler-Brown) and automated (EPANET) exposure models against historical tap water measurements. [file 1476-069X-10-47-S5.DOCX]

# Additional File 5

# Title: Comparison of Exposure Models against Historical Tap Water Measurements

Analyses in the accompanying paper examine the extent to which a plausible increase in accuracy in the exposure model changes the epidemiological results. However because of the importance of exposure estimates we also made comparisons between the manual and automated methods; and between the model estimates and a set of 75 historical water samples. The purpose of the comparisons was to improve our understanding of the exposure assessment models and increase our confidence that they reflected actual PCE exposure.

Because of measurement error and missing information the measured samples were not a gold standard. On the contrary, it is plausible the models are a better representation of exposure than the measurements. However if there were no apparent association between the model and the measurements, this would call into question the validity of the model. There are a number of attributes of the measured samples that are not explicit model parameters (e.g., season, sampling personnel, town, type of sampling point [tap, hydrant, unknown], etc.), some of which might be related to actual PCE levels (e.g., town) while others are sources of measurement error (type of sampling point, sampling personnel). We therefore undertook the comparisons with the objective of shedding further light on the exposure assessment model and how it was operating in this setting.

The distribution of measured concentrations differed by the magnitude of estimated water flow at the sampling location. The water flow at sampling locations have very different estimates depending on the modeling method: the mean modeled flow going past the sampling locations was equivalent to usage by 46 households using the manual flow model versus usage by 80 households using the automated model. As expected, when measured concentrations were categorized by flow rate, the concentrations were higher in areas of low flow, and concentrations were lower in areas of high flow. This pattern was seen for both methods. While most estimated flow rates were similar, in some locations, the manual method estimated lower (n=13) or higher flow (n=10) than the automated method. The automated method likely captured high flow areas more accurately because a higher proportion of undetectable PCE levels were seen in these locations with the automated method as compared to the manual method (83% vs. 60%). Comparisons of measured levels by other sampling characteristics have been described elsewhere [1].

One indication of measurement error in the water sample test results is that historically measured concentrations for the 75 water samples ranged from undetectable (ND) to 2,432 μg/L (detection limit was 0.5 μg/L), but fully half of the samples (37 of 75) had undetectable PCE levels despite the fact that the samples were taken at ACVL pipe sections [4]. However every estimate made by both the manual and automated methods at the measured sampling points that were in the highest exposure category (the top 10%) were, without exception, in the detectable half of the measured samples. This was also true for the upper 20% by the automated method.

When the modeled and measured samples were compared, the automated method was significantly better in signaling detectable PCE in the measured samples at the same point. For the manual method, 65% of the estimates above the median corresponded to historical samples points with detectable PCE (the prevalence of detectable PCE in the historical samples was 50%), while 76% of the estimates by the automated method corresponded to detectable samples. For the 75^th^ percentiles in the manual and automated methods, the proportion of detectable PCE in the historical samples was 84% and 89%, respectively. As noted, for the 90^th^ percentile, all were in the detectable fraction of historical samples by both methods. The inference here is that being in the upper exposure categories by either the manual or the automated method indicates with very high confidence that there was PCE in the drinking water at that point, with evidence that the automated method performed better.

As described in the accompanying article, there was a moderate level of correlation between measured and modeled PCE concentrations using the automated method (Spearman rank correlation coefficient=0.65, p<0.0001). In addition, we found that correlations using the automated method were higher in areas that were difficult to model using the manual method’s flow assumptions (Table S2). These included locations in complex pipe configurations (ρ=0.69 vs. 0.49) and at the beginning/middle of a pipe (ρ=0.65 vs. 0.51). While both methods produced similar correlations in low flow areas (i.e., dead ends), the correlation was higher using EPANET in settings with medium and high flow (ρ=0.56 vs. 0.42 for medium flow and 0.51 vs. 0.40 for high flow). The correlation at tap/spigots was lower using the automated method than the manual method (ρ=0.56 vs. 0.79) but it was higher at hydrants with the automated method (ρ=0.67 vs. 0.37). The interpretation of the latter is hampered by the large number of unknown water fixtures that were sampled (64%). Two towns (Bourne and Sandwich) had much better correlations using the automated than manual method (ρ=0.76 vs. 0.53 for Bourne and ρ=0.83 vs. 0.53 for Sandwich). This was likely due to the high percentage of sampling locations with complex piping in these towns. When we examined the installation year of the pipe, the correlation improved with more recently installed pipe using both methods (Table S3). Correlations between the measured and PCE concentrations modeled with the automated method were better during fall vs. spring, and for personnel Sampler 2 vs. Sampler 1. These results were similar to those from the prior validation (Table S3).

Scatter plots of log_e_ transformed PCE measured and model-generated concentrations show slight differences between automated method (Figure 1) and manual method (Figure 2). Both plots show a line of points at a low measured level, reflecting the samples at undetectable levels that were included in the comparison at 0.25 μg/L, half the detection limit. The manual method had slightly higher predicted concentrations compared to the automated method, as shown by a slight shift to the right in the scatter plots. The difference between modeled concentrations was calculated to see if there was a systematic difference between the methods. The median difference in modeled concentrations at validation sample locations was 3.0 ppb with the manual method predicting higher concentration levels more often. However, this difference was not statistically significant. The overall shift in magnitude of predicted concentrations is unlikely to affect the results of our epidemiological analysis.

1. Spence L, Aschengrau A, Gallagher L, Webster T, Heeren T, Ozonoff D: **Evaluation of a model for estimating tetrachloroethylene exposure from vinyl-lined asbestos-cement pipes.** *Environmental Health: A Global Access Science Source* 2008, **7**.

## Table S2 Table Distribution of Measured PCE Concentrations among All Water Samples and According to Water Flow

|  | **Number** | **Number** | **Percent** |  |  |  |  | |  |  |
| --- | --- | --- | --- | --- | --- | --- | --- | --- | --- | --- |
|  | **of** | **with ND** | **with ND** |  |  | **75^th^** |  | |  |  |
|  | **Samples** | **Level** | **Level** | **Mean** | **Median** | **Percentile** | **Range** | | | |
|  |  |  |  |  |  |  |  |  | |  |
| **All Samples** | 75 | 37 | 49 | 76 | 0.5 | 42 | ND | - | | 2432 |
|  |  |  |  |  |  |  |  |  | |  |
| **According to:** |  |  |  |  |  |  |  |  | |  |
| **Magnitude of Flow according to**  **Automated Exposure Assessment Method*** | |  |  |  |  |  |  |  | |  |
| High (> 66 homes) | 18 | 15 | 83 | 5 | ND | ND | ND | - | | 59 |
| Medium (4-66 homes) | 24 | 13 | 54 | 27 | ND | 44 | ND | - | | 190 |
| Low (< 3 homes) | 33 | 9 | 27 | 151 | 16 | 62 | ND | - | | 2432 |
| **Magnitude of Flow according to**  **Manual Exposure Assessment Method*** | |  |  |  |  |  |  |  | |  |
| High (> 19 homes) | 15 | 9 | 60 | 11 | ND | 20 | ND | - | | 59 |
| Medium (3-19 homes) | 27 | 18 | 67 | 21 | ND | 22 | ND | - | | 190 |
| Low (< 2 homes) | 33 | 10 | 30 | 151 | 13 | 62 | ND | - | | 2432 |

*Categories of modeled flow rate were determined by the tertiles of each distribution.

ND=non-detectable (detection limit=0.5 μg/L)

## Table S3 Spearman Correlation Coefficients (ρ) between Measured and Model-Generated PCE Concentrations for Locations Sampled in 1980

|  | **Automated**  **Exposure Assessment** | | | **Manual**  **Exposure Assessment** | | |
| --- | --- | --- | --- | --- | --- | --- |
|  | **N** | **ρ** | **P-Value** | **N** | **ρ** | **P-Value** |
|  |  |  |  |  |  |  |
| **All Samples Locations** | 75 | 0.65 | <0.0001 | 75 | 0.54 | <0.0001 |
|  |  |  |  |  |  |  |
| **According to:** |  |  |  |  |  |  |
| **Complexity of Pipe Configuration** | |  |  |  |  |  |
| Simple | 27 | 0.54 | 0.004 | 27 | 0.56 | 0.002 |
| Complex | 48 | 0.69 | <.0001 | 48 | 0.49 | 0.0004 |
| **Magnitude of Assessed Flow** |  |  |  |  |  |  |
| High | 18 | 0.51 | 0.03 | 15 | 0.40 | 0.10 |
| Medium | 24 | 0.56 | 0.004 | 27 | 0.42 | 0.03 |
| Low | 33 | 0.52 | 0.002 | 33 | 0.55 | 0.0009 |
| **Position along Pipe** |  |  |  |  |  |  |
| End | 36 | 0.55 | 0.0004 | 37 | 0.52 | 0.0009 |
| Beginning/Middle | 38 | 0.65 | <.0001 | 38 | 0.51 | 0.0010 |
| **Season Sampled** |  |  |  |  |  |  |
| Spring | 63 | 0.66 | <.0001 | 63 | 0.55 | <0.0001 |
| Fall | 12 | 0.71 | 0.01 | 12 | 0.62 | 0.03 |
| **Water Fixture Sampled** |  |  |  |  |  |  |
| Tap or Spigot | 8 | 0.56 | 0.1 | 8 | 0.79 | 0.02 |
| Hydrant | 13 | 0.67 | 0.01 | 13 | 0.37 | 0.20 |
| Unknown | 54 | 0.56 | <.0001 | 54 | 0.48 | 0.0003 |
| **Personnel Conducting Sampling** |  |  |  |  |  |  |
| Sampler 1 | 67 | 0.61 | <.0001 | 67 | 0.46 | 0.0001 |
| Sampler 2 | 7 | 0.77 | 0.04 | 7 | 0.79 | 0.04 |
| Sampler 3 | 1 | - | - | 1 | - | - |
| **Town** |  |  |  |  |  |  |
| Barnstable | 7 | - | - | 7 | - | - |
| Bourne | 16 | 0.76 | 0.0007 | 16 | 0.53 | 0.03 |
| Brewster | 7 | 0.05 | 0.9 | 7 | 0.02 | 1.0 |
| Chatham | 6 | 0.39 | 0.4 | 6 | 0.39 | 0.4 |
| Falmouth | 6 | 0.49 | 0.3 | 6 | 0.49 | 0.3 |
| Provincetown | 5 | - | - | 5 | - | - |
| Sandwich | 9 | 0.83 | 0.006 | 9 | 0.53 | 0.1 |
| Plymouth | 19 | 0.76 | 0.0002 | 19 | 0.83 | <0.0001 |
| **Pipe Installation Year** |  |  |  |  |  |  |
| 1968-1972 | 21 | 0.43 | 0.05 | 21 | 0.50 | 0.02 |
| 1973-1976 | 28 | 0.57 | 0.002 | 28 | 0.55 | 0.002 |
| 1977-1980 | 26 | 0.62 | 0.0007 | 26 | 0.45 | 0.02 |

*Some correlations were not calculated because of the small sample size or because all water samples had undetectable PCE levels.
